# A new analytical framework for missing data imputation and classification with uncertainty: Missing data imputation and heart failure readmission prediction

**DOI:** 10.1371/journal.pone.0237724

**Published:** 2020-09-21

**Authors:** Zhiyong Hu, Dongping Du

**Affiliations:** Department of Industrial, Manufacturing and Systems Engineering, Texas Tech University, Lubbock, TX, United States of America; Universitatsmedizin Greifswald, GERMANY

## Abstract

**Background:**

The wide adoption of electronic health records (EHR) system has provided vast opportunities to advance health care services. However, the prevalence of missing values in EHR system poses a great challenge on data analysis to support clinical decision-making. The objective of this study is to develop a new methodological framework that can address the missing data challenge and provide a reliable tool to predict the hospital readmission among Heart Failure patients.

**Methods:**

We used Gaussian Process Latent Variable Model (GPLVM) to impute the missing values. Specifically, a lower dimensional embedding was learned from a small complete dataset and then used to impute the missing values in the incomplete dataset. The GPLVM-based missing data imputation can provide both the mean estimate and the uncertainty associated with the mean estimate. To incorporate the uncertainty in prediction, a constrained support vector machine (cSVM) was developed to obtain robust predictions. We first sampled multiple datasets from the distributions of input uncertainty and trained a support vector machine for each dataset. Then an optimal classifier was identified by selecting the support vectors that maximize the separation margin of a newly sampled dataset and minimize the similarity with the pre-trained support vectors.

**Results:**

The proposed model was derived and validated using Physionet MIMIC-III clinical database. The GPLVM imputation provided normalized mean absolute errors of 0.11 and 0.12 respectively when 20% and 30% of instances contained missing values, and the confidence bounds of the estimations captures 97% of the true values. The cSVM model provided an average Area Under Curve of 0.68, which improves the prediction accuracy by 7% as compared to some existing classifiers.

**Conclusions:**

The proposed method provides accurate imputation of missing values and has a better prediction performance as compared to existing models that can only deal with deterministic inputs.

## Introduction

The digitized healthcare data has increased significantly due to the wide adoption of electronic health records (EHR) system, which provides useful resources to advance clinical research and improve healthcare [1]. Many studies have been done to extract important information from EHR to predict different events such as readmission and mortality. [1–6]. Despite the vast opportunities, EHR datasets usually contain many missing values, which poses a great challenge on data analysis and clinical applications [7]. One typical approach in dealing with the incomplete data is to delete the instances that contain missing information. However, this is not efficient for EHR data where majority of the values are missing. For example, at a New York academic medical center, 48.9% of patients with disease code for pancreatic cancers did not have corresponding disease documentation in pathology reports [8]. Instead of discarding the missing instances, imputation can be done to estimate the missing information. For instance, a missing value can be replaced with either previous observation (last observation carried forward–LOCF), the average of available data (mean imputation), or fitted values from a regression model (regression imputation).

In the medical literature, many efforts have been made to explore efficient missing data imputation techniques in recent years. Beaulieu-Jones et al. [9] created a representative EHR dataset using a large amount of lab measurements and evaluated the performance of twelve different imputation methods under different missing mechanisms. In their study, two methods, i.e., Multivariate Imputation by Chained Equations (MICE) [10], and softImpute [11], could consistently generate better imputation results as compared with the rest. One possible reason is that these two methods can successfully identify the relationship between different measurements in the constructed dataset, which enables better imputation accuracy. Further, Codella et al. [12] developed an ensemble technique by combining 6 models (i.e., first-order polynomial function, k-nearest neighbors, random forest, perceptron neural network, recurrent neural network architecture with bi-directional gated recurrent unit and bidirectional LSTM) to impute missing values on a time series dataset. Although significant progress has been made to deal with incomplete data, there are still gaps. For example, MICE method assumes a functional relationship between each single variable and all the remaining variables. If the relationship is not properly defined, the method may not provide an accurate estimation of missing values. In addition, Codella’s ensemble technique contains a large number of tuning parameters, and the parameter calibration can introduce extra sources of uncertainty to the imputation (e.g., model errors). In this study, we present a new imputation technique which can overcome the aforementioned limitations and provide a reliable estimation of missing values. Specifically, Gaussian Process Latent Variable Model (GPLVM) will be used to impute the missing values, where a lower dimensional embedding of the observations will be learned from a small complete dataset and then used to impute the missing values in the incomplete dataset.

After imputing the missing values, the complete dataset can be applied to develop models to support clinical decision-making. For example, statistical and Machine Learning (ML) models can be used to predict 30-day readmission of Heart Failure (HF) patients after discharged from Intensive Care Units (ICUs). However, the imputation inevitably introduces uncertainties (e.g. imputation errors) to the dataset. Most ML techniques are sensitive to data uncertainty; and small variations in the inputs can lead to significant changes in model outputs. It is important to quantify these uncertainties to ensure accurate modeling and decision-making. Support Vector Machine (SVM) is one of the most popular models in ML field due to its superior performance on high dimensional data and the capability of handling nonlinear classification. But traditional SVM cannot deal with data uncertainty and is sensitive to noises. In this study, we designed a constrained SVM (cSVM) to account for the data uncertainty (imputation errors). Specifically, multiple datasets were constructed by sampling from the imputed distributions, which were then used to train a group of SVMs. Further, an optimal classifier is identified by selecting the support vectors that maximize the separation margin of a newly sampled dataset and eliminate the underperformed support vectors due to data uncertainty.

The proposed framework was used to predict the 30-day readmission of HF patients using the MIMIC-III database. Heart disease is the leading cause of death and about 5.7 million adults in the United States have HF [13, 14]. Hospitalization provides patients with optimized treatment to reduce mortality and improve treatment outcomes. A continued challenge in the care of HF patients is the high readmission rate. The 30-day readmission rate for HF is more than 20% and the 6-month readmission rate is up to 50% [15]. It is important to predict the readmission rate before discharges so care providers can be proactive and take actions to reduce readmission risk rather than responding to its consequences. Some models have been developed to predict the HF readmission, but the results are suboptimal. Ross et al. [16] reviewed the models for HF readmission prediction between 1950 and 2007 and reported a best C-statistic of 0.60. Frizzell et al. [17] compared 4 ML algorithms for the prediction of 30-day HF readmission and the best C-statistic is 0.624. Awan et al. [18] developed a multi-layer perceptron-based approach for the prediction of 30-day HF readmission, which provide a C-statistic of 0.62. HF readmission prediction needs to be improved and more research efforts are demanded to explore accurate models. The objective of this study is to address the challenges of missing data associated with EHR data analysis and readmission risk prediction.

The rest of the paper is organized as follows. Descriptions of the dataset and clinical variables used to test the methodological framework are presented in the Materials and Methods section. The GPLVM based imputation and cSVM are developed in the section of Readmission Prediction Model. The missing data imputation and readmission prediction performance are analyzed and compared with existing methods in Results, which are followed by Discussions and Conclusions sections.

## Materials and methods

### Dataset

The HF readmission data was constructed from the publicly available MIMIC-III database [19], which consists the EHR data for over 40,000 patients in the critical care unit of the Beth Israel Deaconsess Medical Center from 2001 to 2012. We first discarded patients with age under 18 at admission and died in the ICU. Then patients with discharge diagnosis as HF were screened out based on the International Classification of Diseases, 9^th^ Revision codes (ICD-9) 402.01, 402.11, 402.91, 404.01, 404.03, 404.11, 404.13, 404.91, 404.93, 428.xx [20]. In total, we got 5959 patients with 8439 ICU stays, among which there were 1000 (11.8%) 30-day readmissions. The data will be used to train the models and conduct cross-validation where 80% will be used as training and 20% as testing. The code for data extraction was developed based on the open source code (https://github.com/Jeffreylin0925/MIMIC-III_ICU_Readmission_Analysis) from Lin et al. [3].

### Data preprocessing

A few clinically important features were taken out from the dataset to build the classifier, including demographics, chart events, and length of stay (LOS). Among these data, demographic variables were widely used in risk stratification models, which provide basic information such as gender, age, race. Chart events are time series data, which represent the variation of patients’ physiological conditions during their stay. LOS is the time in hours that a patient spends during the stay. Table 1 provides a summary of these variables for the whole cohort, readmitted group, and non-readmitted group, where continuous variables are expressed as median (interquartile range) while categorical variables are expressed as percentages. All these features were used to develop a classifier to predict the readmission of HF patients.

**Table 1:** Summary of data.

Features	Total (N = 8439)	Readmitted Group (N = 1000)	Non-readmitted Group (N = 7439)
**BUN mean**	27.00 (18.00, 42.00)	29.50 (19.75, 46.50)	26.44 (17.67, 41.50)
**Creatinine mean**	1.20 (0.85, 2.35)	1.40 (0.93, 2.95)	1.20 (0.85, 2.27)
**DSP mean**	57.96 (51.87, 65.20)	58.02 (51.67, 65.22)	57.95 (51.91, 65.20)
**FIO mean**	0.50 (0.40, 0.58)	0.47 (0.40, 0.55)	0.50 (0.40, 0.58)
**Glucose mean**	128.69 (110.89, 155.3)	129.78 (109.9, 158.4)	128.60 (111, 155)
**HR mean**	80.60 (71.68, 90.37)	80.85 (72.89, 91.13)	80.57 (71.52, 90.25)
**MBP mean**	75.40 (69.23, 83.14)	74.41 (67.66, 83.09)	75.48 (69.44, 83.14)
**OS mean**	96.68 (95.44, 97.87)	96.91 (95.63, 98.08)	96.65 (95.43, 97.83)
**RR mean**	19.38 (17.20, 22.00)	19.67 (17.37, 22.79)	19.36 (17.20, 21.91)
**SBP mean**	119.23 (108.84, 132.1)	116.98 (105.9, 131.2)	119.60 (109.3, 132.2)
**Temperature mean**	36.65 (36.36, 36.94)	36.58 (36.29, 36.86)	36.66 (36.37, 36.95)
**Weight mean**	80.48 (67.43, 96.00)	79.87 (67.84, 95.15)	80.55 (67.40, 96.10)
**pH mean**	7.37 (6.95, 7.42)	7.37 (7.00, 7.43)	7.37 (6.94, 7.42)
**BUN std**	1.73 (0.58, 3.58)	2.05 (0.71, 4.02)	1.73 (0.58, 3.54)
**Creatinine std**	0.07 (0.00, 0.17)	0.07 (0.00, 0.21)	0.07 (0.00, 0.17)
**DSP std**	9.44 (7.47, 11.94)	9.42 (7.43, 11.79)	9.45 (7.47, 11.95)
**FIO std**	0.05 (0.00, 0.19)	0.04 (0.00, 0.14)	0.06 (0.00, 0.19)
**Glucose std**	25.16 (13.91, 41.68)	25.79 (14.90, 42.21)	25.08 (13.72, 41.60)
**HR std**	7.04 (5.11, 9.56)	6.82 (5.06, 8.99)	7.07 (5.12, 9.65)
**MBP std**	9.69 (7.76, 12.14)	9.57 (7.59, 12.25)	9.70 (7.78, 12.12)
**OS std**	2.04 (1.57, 2.75)	1.99 (1.49, 2.71)	2.05 (1.58, 2.76)
**RR std**	3.56 (2.92, 4.43)	3.67 (3.01, 4.58)	3.55 (2.91, 4.41)
**SBP std**	13.09 (10.42, 16.41)	12.58 (9.83, 15.90)	13.16 (10.54, 16.48)
**Temperature std**	0.40 (0.29, 0.53)	0.39 (0.29, 0.51)	0.40 (0.29, 0.53)
**Weight std**	0.07 (0.00, 0.71)	0.05 (0.00, 0.56)	0.07 (0.00, 0.71)
**pH std**	0.02 (0.00, 0.05)	0.01 (0.00, 0.05)	0.02 (0.00, 0.05)
**Age**	73.39 (62.70, 81.99)	72.74 (61.58, 81.70)	73.49 (62.87, 82.04)
**LOS**	63.17 (34.33, 118.4)	72.09 (41.09, 147.9)	61.57 (33.79, 115.8)
**Gender**			
Female	46.19	43.20	46.59
Male	53.81	56.80	53.41
**Race**			
Asian	6.45	3.70	6.82
Black	12.32	18.70	11.47
Hispanic	74.32	70.40	74.85
Other	6.91	7.20	6.87
**Insurance type**			
Government	1.58	1.00	1.65
Self-pay	74.83	77.50	74.47
Medicare	17.55	14.00	18.03
Private	6.04	7.50	5.85
**Discharge location**			
Home	15.95	12.80	16.37
Home health care	29.79	26.70	30.21
Long-term care hospital	8.63	14.60	7.82
Rehab hospital	16.34	16.40	16.33
SNF	26.32	27.60	26.15
Other	2.97	1.90	3.12

std: Standard Deviation.

#### Demographics

Five demographic attributes were extracted including gender, age, race, insurance type and discharge location. The first three features are frequently used in readmission risk prediction in the literature. Since HF hospitalization is highly correlated with medical costs, different insurance types might affect patient’s decision for discharge and readmission. As for discharge location, patients would receive different health care at different discharge facilities, which might potentially influence their recovery and thus affecting the readmission rate. All features except age were coded with dummy variables for model development.

#### Chart events

Chart events are measurements of patients’ vital signs, such as heart rate and blood pressure, during their stay in the ICU. Although the data contains a significant amount of valuable information, they have not been explored much in risk prediction [3]. It has been reported that the charted events within the 48 hours before patients’ discharge are correlated with the readmission rate [21, 22]. In this study, 13 numerical attributes in 48 hours prior discharge were used, which include Blood urea nitrogen (BUN), Creatinine, Diastolic blood pressure (DSP), Fraction inspired oxygen (FIO), Glucose, Heart rate (HR), Mean blood pressure (MBP), Oxygen saturation (OS), Respiratory rate (RR), Systolic blood pressure (SBP), Temperature, Weight and pH. Two basic statistical parameters, i.e. mean and standard deviation, were calculated based on the measurements of each variable within the last 48-hour window. The corresponding features were considered as missing when no measurements were available in the 48-hour window.

### Readmission prediction model

As aforementioned, missing values are prevalent in the EHR, which poses a great challenge on readmission risk prediction. To facilitate an accurate prediction of readmission, we will first perform missing data imputation using GPLVM. This model can provide a mean estimate of each missing feature along with the variance quantifying the imputation error. Further, the imputation error will be propagated into the cSVM model to achieve a robust risk prediction. The imputation and prediction models are described in detail as follows.

#### Gaussian Process Latent Variable Model (GPLVM)

GPLVM is an extension of Gaussian Process (GP) [23], where the functional relationship between inputs and outputs is assumed to have a GP prior, and the inputs are treated as latent variables to be estimated along with model hyperparameters [24]. To better understand GPLVM, we will first review the concepts of GP. Let $Y=[y_{1},y_{2},\ldots ,y_{M}]\epsilon R^{N\times M}$ denote the complete observations, i.e. no missing values, where *N* is the total number of patients and *M* is the total number of attributes. In the general GP modelling framework, the input $X={\left[x_{1},x_{2},\ldots ,x_{N}\right]}^{T}\epsilon R^{N\times Q}$ is known and each dimension of the data is modelled as

$$y_{m}=f_{m}+e_{m}$$ (1)

where **f**_*m*_ = [*f*_*m*_(***x***_1_),*f*_*m*_(***x***_2_),…,*f*_*m*_(***x***_*N*_)]^*T*^ is the function value at each input, $e_{m}∼N(0,\tau^{2}I_{N})$ is the observation noise. Here *f*_*m*_(∙) is a GP, that is

$$f_{m}(x)∼GP(\mu (x),k(x,x^{\prime }))$$ (2)

where *μ*(***x***) is the mean function and *k*(***x***,***x***′) is the covariance function. Therefore, the conditional distribution of ***y***_*m*_ given *X* is

$$y_{m}|X,\theta ∼N(\mu_{m},K_{NN}+\tau^{2}I_{N})$$ (3)

where ***μ***_*m*_ = [*μ*_*m*_(***x***_1_),*μ*_*m*_(***x***_2_),…,*μ*_*m*_(***x***_*N*_)]^*T*^ is the mean of ***y***_*m*_, *K*_*NN*_ is the covariance matrix with *K*_*ij*_ = *cov*(*f*_*m*_(***x***_*i*_),*f*_*m*_(***x***_*j*_)), ***θ*** groups all the parameters to be estimated. By taking all the attributes together, *Y* should be modelled as a multiple-output GP. Further, we assume conditional independence across different attributes, then the likelihood of the observations can be written as

$$p(Y|X,\theta )=\prod_{m=1}^{M}p(y_{m}|X,\theta )$$ (4)

which can be used to estimate the model parameters through Maximum Likelihood Estimation. However, when the input *X* is unknown, it should be estimated along with the hyperparameters.

To obtain the distribution of the latent variable *X*, we seek to optimize the likelihood of data (to simplify the notation, the dependence over ***θ*** was omitted)

p(Y)=∫p(Y|X)p(X)dX (5)

However, the nonlinear incorporation of *X* inside the kernel function makes the optimization intractable. Thus, a variational distribution *q*(*X*) approximating the true posterior distribution *p*(*X*|*Y*) is introduced to derive the following variational lower bound for *log*{*p*(*Y*)} [25]

$$L(q)=\int q(X)log\{p(Y|X)\}dX-\int q(X)log\frac{q(X)}{p(X)}dX$$ (6)

where the second term is the KL divergence between the variational distribution and the prior distribution of *X*, which could be analytically calculated. Further, the first term in Eq 6 can be written as

$$l(q)=\int q(X)\;\log\left\{p(Y|X)\right\}\;dX=\sum_{m=1}^{M}\int q(X)\;\log\left\{p(y_{m}|X)\right\}\;dX$$ (7)

where its lower bound can be derived using the variational sparse GP regression method. This lower bound can then be used to estimate the model parameters ***θ*** and latent variable *X*.

When the model parameters and latent variables are identified, the GPLVM can be used to estimate missing values. Let $z_{*}=[z_{*}^{O},z_{*}^{U}]\in R^{N}$ be a row vector representing the measurements of a single patient, where $z_{*}^{O}\in R^{N_{o}}$ and $z_{*}^{U}\in R^{N_{u}}$ denote the observed and missing values respectively. The imputation is done as follows. Firstly, the distribution *q*(x_*_) of the latent variable x_*_ corresponding to z_*_ will be obtained by maximizing the following density [26]:

$$p(z_{*}^{O}|Y)=\frac{\int p(z_{*}^{O},Y|X,x_{*})p(X,x_{*})dX}{\int p(Y|X)p(X)dX}$$ (8)

Let $h_{*}=[h_{*}^{O},h_{*}^{U}]\in R^{N}$ be the latent function value corresponding to z_*_, then the density of the $h_{*}^{U}$ can be calculated:

$$q(h_{*}^{U})=\int p(h_{*}^{U}|x_{*})q(x_{*})\;dx_{*}$$ (9)

Although the marginalization of x_*_ generates an intractable multivariate density, the moments of the density $q(h_{*}^{U})$ are computable for some covariance functions. Therefore, the mean and variance of the missing part $z_{*}^{U}$ could be calculated as $E(z_{*}^{U})=E(h_{*}^{U}),\;cov(z_{*}^{U})=cov(h_{*}^{U})+\tau^{2}I_{N_{u}}$, where the mean provides the estimate of missing values and the variance quantifies the uncertainty associated with the mean estimate. This information will be further propagated into the classification model described in the next section to obtain a robust estimation of readmission risk.

To evaluate the performance of the imputation method, two metrics were used. The first metric is Mean Absolute Error (MAE), which is calculated as: $\frac{1}{N_{miss}}\sum_{(i,j)\in M}\left|\pi_{ij}-y_{ij}\right|$, where *N*_*miss*_ is the number of missing values, M contains the index for all the missing values, *π*_*ij*_ and *y*_*ij*_ respectively represent the imputed mean and the true value. Since GPLVM can also provide the estimation of variance, another metric calculates the proportion of the true values that were contained in the confidence bound *π*_*ij*_±2*σ*_*ij*_.

#### Constrained Support Vector Machine (cSVM)

SVM is a well-established classifier in the ML community, which has been widely applied in different applications [27]. However, traditional SVM can only deal with deterministic inputs. When there is uncertainty involved, SVM needs to be modified to consider the uncertainty in both training and validation processes. In this study, the GPLVM model provides statistical estimations of missing values and quantifies the uncertainty (e.g., imputation error and measurement noises) in all patients’ data. To propagate the uncertainty into classification, we developed a constrained SVM model, which is described as follows.

Given the data $D={\left\{s_{j},y_{j}\right\}}_{j=1}^{N},\;y_{j}\in \{-1,1\}$, $s_{j}\in R^{M}$, the original SVM solves the following problem to maximize the margin between two classes [28]:

$$\underset{w,b,\xi_{j}}{\min}\frac{1}{2}w^{T}w+C\sum_{j=1}^{N}\xi_{j}$$ (10)

$$\begin{array}{c} s.t.\;y_{j}[w^{T}\varphi (s_{j})+b]\geq 1-\xi_{j}\\ \xi_{j}\geq 0 \end{array}$$

where ***w*** is the weight vector, *b* the bias, *C* is a term determining the cost of mis-classification, *ξ*_*j*_ is a slack variable, *φ*(∙)is a kernel function mapping the original inputs to the feature space. SVM aims at selecting some points from each class to construct a hyperplane that could best separate the two classes. The selected points are called support vectors and the performance of SVM greatly depends on the support vectors [28]. When there are uncertainties involved, the hyperplane could be affected greatly, thus influencing the classification performance of SVM. For example, the imputed values in this study are described as probability distributions, and the true values are random variables that can take any values with a certain probability. Different samples of the missing values can lead to different classification models and provide different classification results, i.e., readmission or non-readmission. Therefore, it is important to consider the input uncertainty to achieve a robust prediction.

To account for input uncertainty, we will first sample *p* sets of data from the distributions of missing values. Then, a ***w***_*i*_ is trained with each set of samples, which can generate *p* sets of weighting vectors ${\left\{w_{i}\right\}}_{i=1}^{p}$. Further, a constrained SVM model is trained using ${\left\{w_{i}\right\}}_{i=1}^{p}$ and a new set of samples by solving the following optimization problem

$$\underset{w,b,\xi_{j}}{\min}\frac{1}{2}w^{T}w-\lambda \sum_{i=1}^{p}w^{T}w_{i}+C\sum_{j=1}^{N}\xi_{j}$$ (11)

$$\begin{array}{c} s.t.\;y_{j}[w^{T}\varphi (s_{j})+b]\geq 1-\xi_{j}\\ \xi_{j}\geq 0 \end{array}$$

where *λ* is a penalty coefficient. The penalty term containing ***w***_*i*_s can incorporate the uncertainty information in the data into SVM optimization. Specifically, minimizing the similarity between ***w*** and ***w***_*i*_s will guide the optimization to seek a ***w*** that can maximumly capture the variations across all sets of data. Since direct solution of this problem is complex, the following dual problem was formulated based on the Karush-Kuhn-Tucker conditions [29]:

$$\underset{\alpha_{j}}{\max}\sum_{j=1}^{N}\alpha_{j}-\frac{1}{2}\sum_{j=1}^{N}\sum_{k=1}^{N}\alpha_{j}\alpha_{k}y_{j}y_{k}{\varphi (s_{j})}^{T}\varphi (s_{k})+\lambda \sum_{j=1}^{N}{\left(\sum_{i=1}^{p}w_{i}\right)}^{T}\alpha_{j}y_{j}\varphi (s_{j})$$ (12)

$$\begin{array}{c} s.t.\;0\leq \alpha_{j}\leq C\\ \sum_{j=1}^{N}\alpha_{j}y_{j}=0 \end{array}$$

which is a quadratic programing problem and can be solved using standard optimization packages. Compared with the original SVM, cSVM includes a penalty term to consider the support vectors identified from the classifiers trained with multiple samples. This will take into account the uncertainty in the imputed dataset. Later we will show that this penalty term will improve the performance of classification in the presence of uncertainty (i.e., missing values) (See Results Section).

## Results

### Feature selection

To avoid overfitting, feature selection was performed on the imputed dataset to select the attributes that are strongly correlated with the outputs. This procedure was done for all the variables list in in the Data Preprocessing section. Logistic regression (LR) with LASSO regularization was used to select the significant features for subsequent analysis. The selected features include the means of BUN, DBP, FIO, Glucose, HR, RR, SBP, temperature, weight, pH, and the standard deviations of FIO, HR, MBP, OS, RR, SBP, temperature, weight, as well as age, LOS, GCS eye, GCS verbal, Gender, Race, Insurance and Discharge location.

### Missing data imputation

When a vital sign recording is not available within the 48 hours before discharge, the corresponding statistical features are missing and need to be imputed through GPLVM. The original dataset used in this study contains 8439 instances, and 88% of them have missed at least one vital sign recording. To test the performance of the missing data imputation, we extracted all the instances (i.e. patients) with complete statistical features from the original data, and deliberately set some items as missing under the missing at random assumption [9]. The incomplete datasets were generated following three steps: firstly, for each feature, a linear regression model was fitted using the remaining features, and the quartiles of each fitted feature was calculated; secondly, different proportion of instances, i.e., 20% and 30%, were randomly selected; lastly, within each selected instance, the feature value was set as missing if its value was within the predetermined quartile range of its fitted value (e.g. between 2^nd^ and 3^rd^ quartile). GPLVM with a squared exponential kernel function was trained using complete instances and tested the imputation performance on the generated incomplete dataset.

**Fig 1:**
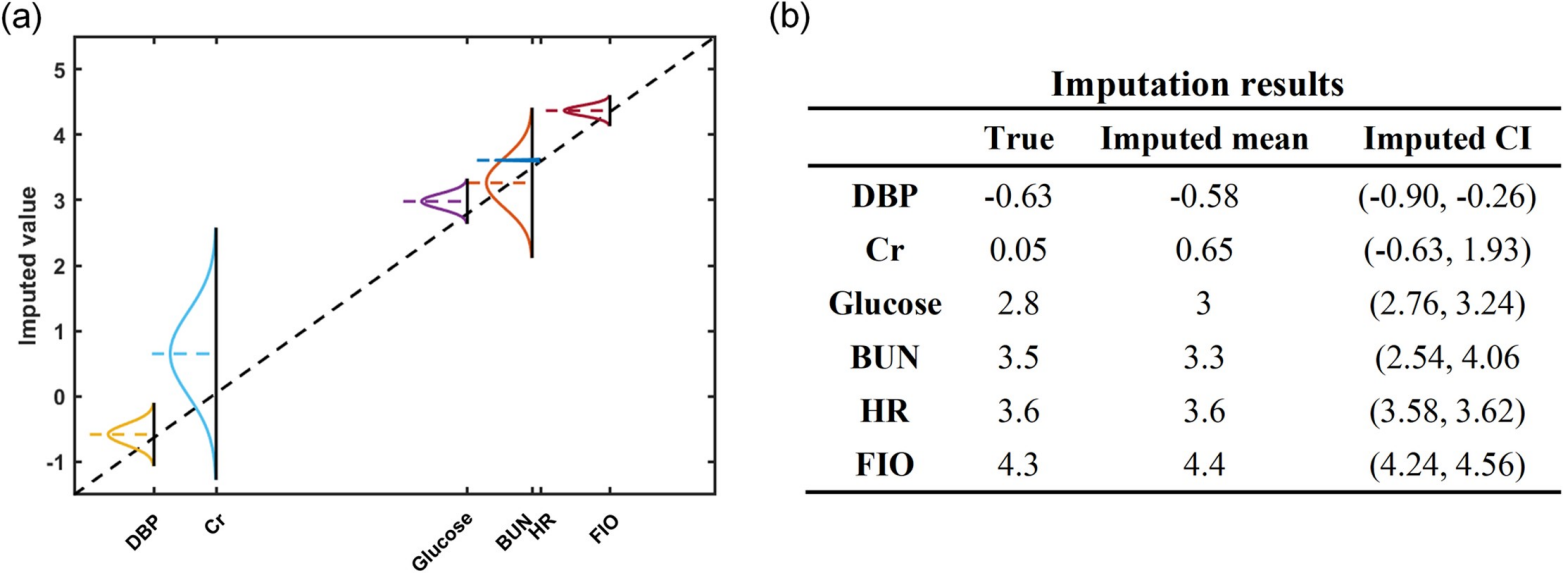
Imputed results of a single instance.

To provide some insights into the GPLVM-based imputation results, the imputation for a patient with six missing values: BUN, Creatinine, DSP, FIO, Glucose, and HR is demonstrated in Fig 1. In Fig 1(A) the vertical dark solid lines mark the true measurements while the horizonal dashed lines represent the imputed means. When the intersection between these two lines lies on the black dashed line, the mean estimate matches well with the true value. As seen in Fig 1(A), five missing values were well imputed with the mean estimates except for Cr. The true values and the imputed means are also shown in Fig 1(B). The advantage of GPLVM based imputation is that it can provide the variance along with the mean estimate, which is displayed as the probability density function in Fig 1(A) and the confidence interval of the imputation (CI, bounds within 2-fold standard deviation) in Fig 1(B). For Cr, the true value is away from the estimated mean, but it is still within imputation bounds.

**Fig 2:**
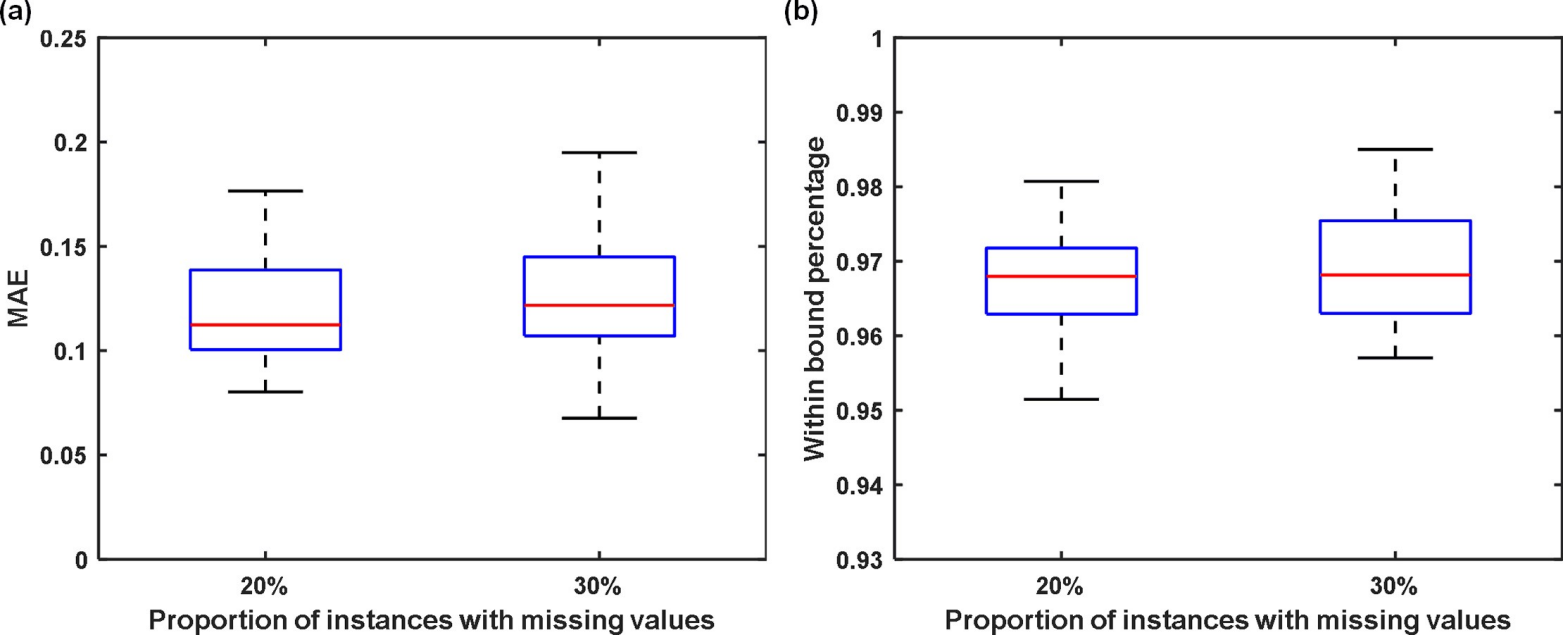
Imputation performance. (a) Mean absolute error (MAE) of the imputation when 20% and 30% of the instances contain missing values, and (b) proportion of true values that were contained in the imputation confidence bounds when 20% and 30% of the instances have missing values.

To quantify the imputation accuracy, we normalized all variables to a common scale of zero mean and unit standard deviation. The two metrics, i.e. MAE and the proportion of the true values captured by the imputation bound, were used to evaluate the imputation accuracy. Since the incomplete dataset is generated randomly, experiments were repeated for 50 times, and the box plots of MAEs for different percentages of instances that have missing values are shown in Fig 2(A). As seen, the GPLVM can impute all missing values at an average MAE of 0.11 and 0.12 for 20% and 30%. In addition, when the proportion of missing values increases, the variance of MAE increases. In addition, we also looked at how many true values are within two standard deviation of the mean estimates, i.e., (*π*_*ij*_+2*σ*_*ij*_). As seen in Fig 2(B), about 97% of the true values are within two standard deviation of the mean estimates. In other words, 97% of the estimated confidence bounds contain the true values of missing data. The metric is important because when the true values of missing data are within the confidence bounds, they will be accounted in the cSVM, thus the classification will be more robust to the uncertainty introduced by missing data imputation.

### Model performance

Since missing values are considered as random variables in the GPLVM imputation, traditional ML methods, such as SVM, cannot take such variables as inputs. To make full use of the information, a new classifier, cSVM, was trained for readmission prediction. Since the data is highly unbalanced, patients with no-readmission were down-sampled to obtain an even training data, i.e., the ratio between the two classes is 1:1. Considering the nonlinearity of the data, the radial basis function (RBF) kernel was used to train the classifier. We investigated four parameters to optimize the performance of cSVM. These parameters include box constraint *C*, kernel scale *σ*, number of pre-sampled datasets *p*, and penalty coefficient *λ*. Specifically, *σ* was set as the optimal value that provides the best performance for SVM. In addition, we found that the accuracy of cSVM is not significantly sensitive to the sample size *p*. We recommend choosing a sample size large than 30. However, *p* should be determined case-by-case based on the number of features that involve uncertainty. In our study, we used *p* = 200. Further, *λ* is set to be 1 and the box constraint is set to be 10000.

**Fig 3:**
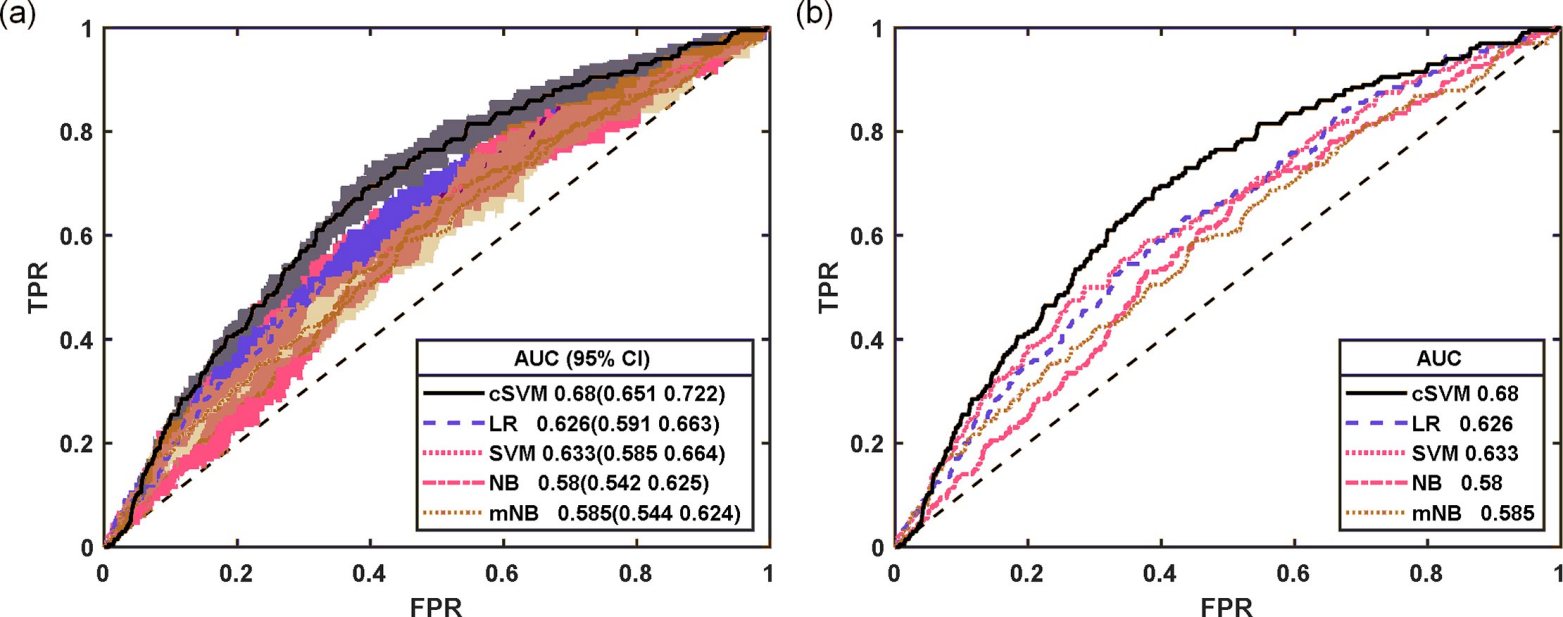
AUC of different classifiers.

To benchmark the results of the proposed cSVM model, we trained different classifiers of LR, Naïve Bayes (NB), traditional SVM, and a modified NB (mNB) which can deal with input uncertainty [30]. The receiver operating characteristic (ROC) curves of different classifiers are plotted in Fig 3. The left figure shows the ROC curves and their 95% bootstrap confidence bounds, and the right figure shows the average ROC curve. As seen in the figure, cSVM shows a better ROC as compared to the other four classifiers and the area under curve (AUC) of cSVM is the largest. cSVM has a 7% improvement on AUC as compared to the traditional SVM, which demonstrates that the introduced penalty term could improve the classification performance. Further, the mean True Positive Rates (TPRs) with respect to different False Positive Rates (FPRs) for each classifier were provided in Table 2, which also reveals that cSVM could consistently achieve better results compared with other classifiers. In addition to the AUC, some targeted operating points are also useful in clinical applications [3], for example application of high-sensitivity (TPR) can rule out the disease, whereas high-specificity (1-FPR) can rule in the disease [31]. Thus, the operating points with sensitivity and specificity fixed at 0.85 and 0.85 were adopted to evaluate the performance of different classifiers [32, 33], and results are given in Table 3. From the table, we can find that, compared with LR, NB, mNB and SVM, cSVM can improve the performance by 18%, 61%, 57% and 27% at operating point of high-sensitivity and by 22%, 63%, 35%, 6% at the operating point of high-specificity. All the classifiers are trained with the complete dataset after the GPLVM-based missing data imputation. The LR, NB and SVM models used the mean estimates of the imputed data.

**Table 2:** TPR with respect to different FPR.

FPR Classifier	0.1	0.2	0.3	0.4	0.5	0.6	0.7	0.8	0.9
**LR**	0.180	0.355	0.460	0.590	0.665	0.760	0.855	0.910	0.960
**NB**	0.135	0.250	0.375	0.535	0.640	0.725	0.800	0.865	0.940
**mNB**	0.182	0.303	0.414	0.505	0.601	0.707	0.803	0.869	0.934
**SVM**	0.215	0.381	0.500	0.595	0.665	0.754	0.835	0.915	0.965
**cSVM**	**0.249**	**0.410**	**0.570**	**0.695**	**0.765**	**0.835**	**0.885**	**0.920**	**0.970**

**Table 3:** Performance comparison at operating points corresponding to high sensitivity and specificity.

Classifier	Specificity (95% CI)	Sensitivity (95% CI)
**LR**	0.311 (0.280, 0.330)	0.275 (0.223, 0.364)
**NB**	0.229 (0.208, 0.248)	0.205 (0.149, 0.258)
**mNB**	0.234 (0.213, 0.261)	0.248 (0.187, 0.321)
**SVM**	0.290 (0.263, 0.313)	0.315 (0.267, 0.377)
**cSVM**	**0.368 (0.338, 0.398)**	**0.335 (0.288, 0.399)**

Further, to investigate the performance of the combination of GPLVM-based imputation and the cSVM classifier, we replaced the GPLVM with other imputation techniques, and then performed classification using LR, NB and SVM models. Two different imputation methods were used to impute the missing values, which were LOCF and mean imputation. The AUCs of different combinations are given in Table 4. As shown in the table, all three classifiers (LR, NB, and SVM) achieve better AUCs with GPLVM imputation than the LOCF and the mean imputation. This reveals that the GPLVM-based imputation outperforms the traditional LOCF and mean imputation thus benefiting the classification for readmission prediction. The comparison study suggests that the proposed analytical framework improves the readmission prediction performance as compared with traditional methods.

**Table 4:** AUC using different combinations of classifiers and imputation methods.

Classifier	LOCF imputation	Mean imputation	GPLVM imputation
**LR**	0.604 (0.549, 0.635)	0.606 (0.547, 0.644)	0.626 (0.591, 0.663)
**NB**	0.590 (0.550, 0.630)	0.562 (0.513, 0.592)	0.580 (0.542, 0.625)
**SVM**	0.613 (0.581, 0.675)	0.610 (0.574, 0.672)	0.633 (0.585, 0.664)
**mNB**	/	/	0.585 (0.544, 0.624)
**cSVM**	/	/	**0.680 (0.651, 0.722)**

## Discussion

The wide adoption of EHR system provides a great opportunity to mine the large database of patients and analyze patient traits, cause of disease, treatment effects, risks and mortalities. However, missing data is prevalent in EHR system, which poses a great challenge on data analysis to support clinical decision-making. This study developed a new analytical framework to address the missing data challenge and facilitate a robust prediction of HF readmission using EHR data. Specifically, GPLVM was introduced to impute the missing values in EHR dataset. The intuition of using this technique is that physiological measurements are indicators of patient’s condition; and there should be inherent correlations among features of an individual patient and across the patient population. Since GPLVM employs the versality of GP in representing a complex functional relationship between inputs and outputs, the optimized latent variables can provide better representation of the features, which in turn facilitate accurate missing data imputation. In addition, GPLVM can provide both the mean estimates of the missing values as well as their variances that quantify the uncertainty associated with the mean estimate. Quantifying the uncertainty of the imputation is useful, as this information can be propagated into the subsequent analysis to ensure a robust prediction. To propagate the uncertainties (i.e., imputation errors) in the imputed dataset into modeling, a new classifier, cSVM, was developed based on SVM. Different sets of features were randomly sampled from the distributions of missing values, and SVM models were trained using these random samples. A penalization term was introduced to constrain the deviation of the optimal classifier from the group of pre-trained support vectors; this will ensure the optimal classifier is not biased due to the imputation uncertainty. The resulting classifier can retain the discriminative features in the dataset while minimizing the influence of data uncertainty, and thereby improving the classification performance.

The proposed missing data imputation and classification framework was tested and validated using a clinical database to predict 30-day readmission of HF patients discharged from ICUs. The model can provide a better AUC as compared to other popular classifiers in the literature. The improvement in the prediction accuracy is awarded by the better missing data imputation performance of GPLVM and the robust classification of the cSVM. However, further improvement is limited by the efficiency of the predictors (features). Existing studies on 30-day readmission prediction for HF patients have AUCs up to 0.62 [16–18, 34]. A recent study using structured data, such as age and race, and unstructured data, such as discharge notes, to predict the readmission of Congestive Heart Failure patients can achieve an AUC of 0.97 [35], but the model has a high level of complexity and needs extensive validation. The generally low accuracy of readmission prediction can be due to the limited predictive potentials of the features. Future research should be done to discover significant risk factors and biomarkers that can improve HF readmission prediction.

The GPLVM imputation technique learns the correlations among features to estimate missing values. It can provide better estimations for datasets that have correlated attributes. However, for datasets where all attributes are independent, the accuracy of GPLVM needs further validation. In addition, this study calculated the statistical features, i.e., mean and standard deviation, from the vital sign data, and then imputed the missing features using GPLVM. The two statistical features may not be able to exploit all useful information in the time series data, thus the advantage of GPLVM was not fully explored. Future work can be done to model the time series to extract more informative features for readmission prediction.

## Conclusions

Mining EHR datasets to facilitate clinical decision-making has become a popular topic. However, EHR often contains a large amount of missing values, which greatly challenges existing techniques. This study designed a new analytical framework based on the GPLVM and cSVM. The former can effectively impute the missing values in EHR and provide both the mean and variance estimates, while the latter provides better diagnostic capability by incorporating the input uncertainties during model development. The proposed method was tested using the MIMIC-III database and showed better performance for HF readmission prediction compared with some existing models.
